# Clarifying the interpretation of subdistribution hazard ratios in competing-risk analyses: comment on Riis *et al.*, *Eur Thyroid J.* 2024;13: ETJ230181

**DOI:** 10.1530/ETJ-25-0359

**Published:** 2026-02-10

**Authors:** Mohammad Jay, Antoine Eskander, Lorraine Lipscombe, Rinku Sutradhar

**Affiliations:** ^1^ Division of Endocrinology, Department of Medicine, University of Toronto, Toronto, Canada; ^2^ Institute of Health Policy, Management and Evaluation, University of Toronto, Toronto, Canada; ^3^ Department of Otolaryngology – Head & Neck Surgery, Sunnybrook Health Sciences Centre, University of Toronto, Toronto, Ontario, Canada; ^4^ Women’s College Hospital, Toronto, Canada; ^5^ Cancer Program, ICES, Toronto, Canada; ^6^ Division of Biostatistics, Dalla Lana School of Public Health, University of Toronto, Toronto, Ontario, Canada

**Keywords:** competing risks, Fine–Gray model, subdistribution hazard ratio, thyroid epidemiology

Dear Editor,

We read with interest the article by Riis *et al.*, ‘Hyperthyroidism and the risk of non-thyroid cancer: a Danish register-based long-term follow-up study’ (1). The authors are commended for their rigorous population-based design addressing a clinically relevant question in endocrine epidemiology. Their use of high-quality registries, matching, and sensitivity analyses provides valuable evidence on cancer risk after hyperthyroidism.

We write to clarify a methodological aspect: the interpretation of the subdistribution hazard ratio (SHR) from the Fine–Gray competing-risk model. As these methods gain traction in endocrine research, consistent terminology will help clinicians translate findings accurately.

## Understanding the subdistribution hazard ratio

Riis *et al.* used Fine–Gray models to account for the competing risk of death when estimating associations between hyperthyroidism and subsequent cancer (2). The subdistribution hazard reflects how quickly new cases of the outcome accumulate over time, considering competing events. It is not an instantaneous event rate or probability but instead captures the shape of the cumulative incidence curve without representing absolute risk (3).

The SHR can be interpreted as a modified relative rate, describing how an exposure alters the accumulation of events compared with a reference group (2). This ‘rate’ is adjusted because the Fine–Gray model retains individuals who experience a competing event in the risk set, with weighting adjustments, to model the cumulative incidence function (CIF). The SHR quantifies how rapidly the CIF increases between exposure groups. An SHR > 1 indicates faster accumulation (higher cumulative incidence), while an SHR < 1 indicates slower accumulation (lower cumulative incidence) (4).

## Relation to the proportional hazards and competing event frameworks

Most clinicians are familiar with the Cox proportional hazards model, which estimates how exposures affect the instantaneous event rate (hazard) among individuals who remain event-free. In this model, censoring assumes that they could still experience the event later if follow-up continued. This assumption holds when there is only one possible event type, such as time from thyroid nodule detection to fine-needle aspiration, where competing events such as death are rare.

When competing events can occur first, such as death before cancer development, analyses must account for these competing outcomes because once a competing event occurs, the primary event can no longer happen for that individual (4, 5). In this case, the cause-specific Cox model is the natural extension, where individuals experiencing a competing event are censored and removed from the risk set. The resulting cause-specific hazard ratio (HR) quantifies how an exposure affects the hazard of the outcome among those still at risk, making this model ideal for etiologic questions, such as whether an exposure contributes to disease development (4).

The Fine–Gray model instead retains individuals experiencing competing events in the risk set with reduced weighting. This allows estimation of the subdistribution hazard and its corresponding SHR, which links directly to the CIF, the probability of experiencing the event by a given time while accounting for competing outcomes (5, 6). The Fine–Gray model provides a population-level estimate of how exposure changes the observed probability of the outcome in real-world populations (6).

Because these models answer different questions, they may yield different results. The cause-specific model captures biological effects among survivors, whereas the Fine–Gray model captures overall population probability once competing risks are considered (6). For instance, if hyperthyroidism increases both cancer risk and mortality, many patients may die before cancer develops: the cause-specific HR might rise, yet the Fine–Gray SHR may show little change in cumulative incidence. Both perspectives are informative: the HR for etiologic insight and the SHR for population prognosis (6, 7).

## Directionality and magnitude

The SHR’s interpretive value lies primarily in its direction, not magnitude. It indicates whether cumulative incidence is higher or lower after accounting for competing events, but does not reflect absolute risk. When competing events are rare, SHR and cause-specific HR values may approximate each other (4).

Given the non-trivial death rate in the Riis *et al.* cohort, phrasing such as ‘12% increased risk of cancer’ could be misinterpreted as the event probability (1). A clearer phrasing would be ‘a higher subdistribution hazard’, or ‘a greater cumulative incidence over time after accounting for mortality’. In practice, the SHR should be interpreted for its direction, while the corresponding cumulative incidence at fixed time points conveys the absolute risk – the quantity that matters most to patients.

## Recommendations to enhance interpretability

- **Identify the model type for each estimate.** Specify whether results are from a cause-specific or Fine–Gray model. The current study uses both terms (‘cause-specific’ and ‘Fine–Gray model’ CIFs). Aligning terminology across text, tables, and figures improves clarity.
- **Provide absolute risks alongside relative measures.** Report cumulative incidence curves and absolute event probabilities at clinically relevant time points (e.g. 5 or 10 years).
- **Report both model families when feasible** to provide complementary etiologic and prognostic insights.
- **Avoid cross-outcome or cross-study SHR comparisons** because baseline event rates differ across outcomes and populations.
- **Use CIFs, rather than 1 − Kaplan–Meier curves,** as the latter overestimates incidence when competing events exist.

These practices align with published methodological guidance and are straightforward to implement in large population-based studies (4, 8).

## Clinical and methodological implications

Competing-risk methods are increasingly important in endocrine epidemiology, where patients may experience multiple long-term outcomes, such as cancer, cardiovascular disease, or death (9, 10). Misinterpreting hazard-based measures can lead to erroneous conclusions about causality or prognosis. Recognizing that the SHR pertains to population-level cumulative incidence, not individual risk, improves interpretive accuracy (4, 6).

Riis *et al.* minimized bias through matching, comorbidity adjustment, and sensitivity analyses addressing the Berkson bias (1). Our comments aim to enhance interpretive clarity, not critique the study design.

## Conclusion

Riis *et al.* make an important contribution to understanding malignancy risk after hyperthyroidism. To enhance clarity and impact in future work, we encourage authors and reviewers to i) specify the hazard model used, ii) interpret SHRs primarily for direction unless competing events are negligible, and iii) pair relative measures with absolute cumulative incidence. Adopting these practices will improve transparency, reproducibility, and clinical utility.

## Declaration of interest

The authors declare that there is no conflict of interest that could be perceived as prejudicing the impartiality of the work reported.

## Funding

This work did not receive any specific grant from any funding agency in the public, commercial, or not-for-profit sector.
